# Semantic segmentation of thermal defects in belt conveyor idlers using thermal image augmentation and U-Net-based convolutional neural networks

**DOI:** 10.1038/s41598-024-55864-2

**Published:** 2024-03-08

**Authors:** Mohammad Siami, Tomasz Barszcz, Jacek Wodecki, Radoslaw Zimroz

**Affiliations:** 1 AMC Vibro Sp. z o.o., Pilotow 2e, 31-462 Kraków, Poland; 2 Faculty of Mechanical Engineering and Robotics, AGH University, Al. Mickiewicza 30, 30-059 Kraków, Poland; 3 Faculty of Geoengineering, Mining and Geology, Wroclaw University of Science and Technology, Na Grobli 15, 50-421 Wroclaw, Poland

**Keywords:** U-Net, Convolutional neural networks, Semantic segmentation, Thermal imaging, Conveyor systems, Idlers, Thermal defects, Engineering, Electrical and electronic engineering

## Abstract

The belt conveyor (BC) is the main means of horizontal transportation of bulk materials at mining sites. The sudden fault in BC modules may cause unexpected stops in production lines. With the increasing number of applications of inspection mobile robots in condition monitoring (CM) of industrial infrastructure in hazardous environments, in this article we introduce an image processing pipeline for automatic segmentation of thermal defects in thermal images captured from BC idlers using a mobile robot. This study follows the fact that CM of idler temperature is an important task for preventing sudden breakdowns in BC system networks. We compared the performance of three different types of U-Net-based convolutional neural network architectures for the identification of thermal anomalies using a small number of hand-labeled thermal images. Experiments on the test data set showed that the attention residual U-Net with binary cross entropy as the loss function handled the semantic segmentation problem better than our previous research and other studied U-Net variations.

## Introduction

Accurate segmentation of the overheated idler in thermal or, in other words, infrared (IR) images with complex backgrounds is an important task for performing robotic-based inspection of BC systems. As more samples can be captured by an inspection mobile robot within a fixed time, the challenge associated with image processing tasks also increases. Manual analysis of captured thermal images by experts is not just time-consuming but also requires extreme precision due to the presence of different thermal sources in mining sites that can be wrongly identified as overheated idlers. Robotics-based thermal imaging can enable supervisors to minimize or totally exclude the presence of humans in harsh environments like mining sites^1–13^.

BC systems are common transportation systems for continuous conveying of raw materials at mining sites. The standard lifetime, or the minimum L10 life requirement (the required time in which 10% of the idler bearings will eventually fail), should be 50,000 h, or 5–7 years, but by considering the harsh environmental conditions in mining sites, the minimum life span can be considerably decreased^14–18^. Therefore, regular inspection of BC system idlers is considered an important task for preventing sudden breakdowns in production lines in mining sites^19–22^.

The use of artificial intelligence (AI) methods for CM of BC systems is widespread. AI-aided diagnosis methods can reduce the number of errors that can be caused by human operations and help supervisors identify and localize BC faults on large-scale industrial infrastructure. With the rapid development of AI-aided methods in the CM of industrial systems, deep learning methods achieved remarkable results for different monitoring and fault identification tasks^22–30^.

Application of thermal imaging for identification of thermal anomalies on idlers has proven to be a practical way to localize damaged idlers. Manual analysis of thermal images with complex backgrounds can affect the degree of precision of fault identification procedures in large-scale industrial infrastructures^31^. Furthermore, deep learning-based methods have been proven to be a superior methodology in image segmentation, which can overcome the difficulties of low signal-to-noise ratio, low contrast, and poor quality of thermal images captured by inception robots and unmanned aerial vehicles (UAVs)^32–35^.

In our previous works, we have studied different methodologies that might be used for automatic identification of overheated idlers that have been captured by a mobile robot in real-world scenarios. In^36^ we have proposed an image processing pipeline for improving the overall quality of the captured thermal images. Furthermore, we studied the captured thermal images using histogram analysis techniques for the identification of frames with the signs of the thermal anomalies. The proposed methodology was successful in identifying frames that could contain overheated idlers. However, the proposed method was not sufficient for the identification of thermal defects in thermal images with complex backgrounds. To improve the detection results, in^11^ we have proposed a method based on binary classification of captured thermal images using a CNN architecture. Binary classification of extracted hotspots could help to accurately separate frames where other thermal sources were wrongly segmented as overheated idlers. The proposed method could significantly improve the performance of our CM methodology. However, the accuracy of the classification task was once again limited by the performance of the proposed outlier detection technique in^36^.

To meet the needs of robotic-based CM for large-scale BC systems in mining sites, a new idler CM pipeline is being developed. To improve the correct identification and segmentation of overheated idlers in this paper, semantic segmentation of thermal images was carried out using different U-Net architectures for extracting specific temperature patterns in thermal images.

Different CNN-based architectures have been developed in the past decade, and some of them have received attention due to their performance in different areas such as image classification, speech processing, and robotics^37,38^. These network architectures are becoming the standard choice for researchers to solve novel challenges in different fields of study.

In this study, to first improve the size of the data set, we apply data augmentation techniques to the original data sets. We showed that the use of image augmentation, in which a small number of hand-labeled images could help to transform the data set into a larger one through the pre-processing steps. By doing this, we could improve the overall performance of the studied U-Net architectures. Furthermore, in this paper, we perform a comprehensive analysis of the three different U-Net variants, including base U-Net, attention U-Net, and ARes U-Net, in the semantic segmentation of thermal defects in BC idlers. Through this study, the advantages and similarities of the studied U-Net architecture are discussed, along with the challenges involved in robotic-based data collection in mining sites.

## Related works

Application of thermal imaging in the CM of industrial infrastructure has been the topic of several papers. Different traditional image processing (TIP) methods have been proposed for thermal image segmentation. The proposed methodologies can be categorized into four different groups, namely: region-based^39,40^, fuzzy-based^41^, textural analysis^42,43^ and threshold-based methods^44,45^.

Region-based methods can provide remarkable results for performing segmentation tasks in thermal images, as discussed in^39^. A FAsT-Match algorithm is proposed by authors in^40^ for the segmentation of electrical equipment in thermal images. The authors showed the superiority of their results in comparison to other traditional segmentation methods. However, their proposed method was only successful in segmenting images based on low-level semantic information, which could cause over-segmentation in most cases. Wu et al.^41^ introduced a method based on fast fuzzy c-means algorithms for segmentation of thermal images. In textural analysis methods, different features such as edge, color, texture, and motion are extracted and integrated for correct segmentation of desirable objects within an image^42,43^. In threshold-based methods, an optimal threshold value should be calculated for proper separation of the target (foreground) from other regions (background)^45^. In^44^, the authors proposed a method for the extraction of an optimal threshold value based on the definition of entropy information in a processed thermal image. Moreover, the histogram of gray-scaled thermal images has been processed based on the outlier detection concept in^36^.

The mentioned TIP methods are not always suitable for accurate separation of overlapped objects in thermal images with complex scenes, as they have been developed to distinguish the foreground from the background, not the similar objects from each other^46^. Furthermore, they need to be assisted by supervisors by adjusting the filter parameters for accurate identification of particular colors for each target area^47^. Due to the mentioned reasons, the TIP methods are not a proper solution for automatic segmentation of thermal defects in captured thermal images from industrial infrastructure. To address the mentioned issues, the application of deep learning in object segmentation tasks for image processing purposes has been studied by different researchers in recent years.

In semantic segmentation, pixels are treated as a pixel-level classification problem where each pixel within an image is labeled with a specific category. Semantic segmentation can be performed by using different CNN architectures, such as the U-Net network, which can improve the segmentation results over TIP and machine learning (ML) methods^48,49^.

The U-Net model architecture was mainly developed for the semantic segmentation of biomedical images^50^. However, in recent years, it has been tested for solving the semantic segmentation tasks in different research, including brain tumors and MRI images^51,52^, the cityscapes datasets^53^, road scenes^54^ and other datasets^55–59^. It is worth mentioning that while the application of U-Net models in the semantic segmentation of thermal faults has been discussed in previous studies, it is rarely discussed for thermal fault segmentation in thermal images that are captured from industrial infrastructure in harsh environments.

The base U-Net model is used in image processing for fault identification in thermal images that are captured from photovoltaic (PV) panels. In^60^, researchers proposed a semantic segmentation model based on a U-Net architecture to detect the faulty area of PV panels in thermal images that are captured by an UAV system. This algorithm performed well on segmentation tasks, where the highest Jaccard index of their proposed method was 0.94. However, in this study, a limited number of images have been used through the training process, which could cause overfitting. The authors in^61^ presented a modified U-Net architecture for PV array extraction from complex scenes where the Dice score of the network was 0.95. Inspired by the same problem in^62^ authors studied the U-Net architecture and a classifier’s performance for the identification of PV panel faults. Their studies indicated that the U-Net architecture can successfully identify thermal faults in PV panels. However, their training data sets consist of thermal images that were collected manually by an inspector.

The mentioned research showed the significance of different U-Net architectures for semantic segmentation of targets (faults) in thermal images. However, training the fully CNN models requires a large number of samples along with labels, which can be time-consuming to capture or even impossible due to the nature of the study.

## Material and methods

**Figure 1:**
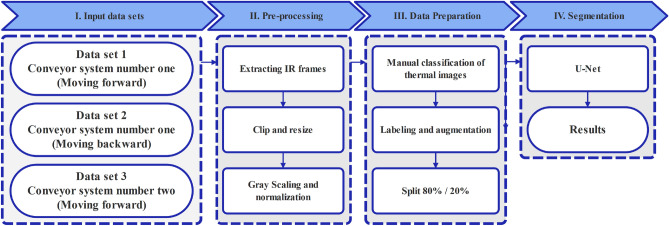
Simplified flowcharts of proposed thermal image processing pipeline for segmentation of overheated idlers in thermal images.

In this section, we first focus on the importance of pre-processing stages for improving the overall quality of the extracted frames from captured thermal videos. The extracted thermal frames undergo different pre-processing stages, including gray-scale transformation and normalization. Furthermore, to improve the segmentation performance of the studied U-Net architecture, different augmentation techniques were applied to extracted frames to increase the size of the training datasets. Afterward, we introduced the studied U-Net architectures in detail. The simplified flowchart of the proposed methodology is described in Fig. 1.

### Pre-processing

Strong noise, uneven brightness, and poor contrast are the general characteristics of the thermal images that are captured in harsh environments. To address the mentioned issue, the raw thermal images need to undergo different pre-processing steps. In the first step, to reduce the complexity of captured scenes and improve the accuracy of segmentation results, colored thermal images are converted to gray-scale 8-bit images. The conversation equation is shown as follows:

1 $$\begin{aligned} I_{\text{ gray } }=0.299 \times I_{red}+0.587 \times I_{\textrm{green}}+0.114 \times I_{blue} \end{aligned}$$

The intensity of RGB channels in an extracted thermal frame can be described as $I_{red}$, $I_{green}$, and $I_{blue}$^63,64^.

To reduce the computation burden and improve the detection results, we defined a 256 $\times$ 256 pixel region of interest on gray-scaled thermal frames^11,36^. Furthermore, to reduce the seasonal changes in the extracted frames and define statistical parameters that work with varying temperature ranges, we normalized the $I_{gray}$. The normalization equation is shown below:

2 $$\begin{aligned} I_{\text{ norm } }=\frac{I_{\text{ gray } }-\mu }{\sigma } \end{aligned}$$

In Eq. (2), $\mu$ refers to the mean value, while $\sigma$ indicates the variance value of the processed gray-scaled thermal frame^65^.

### Data augmentation

Different researchers proposed methodologies for generalizing CNNs with small training data sets. Among the proposed ideas, data augmentation has been considered a useful method for different types of images^66,67^.

Data augmentation techniques can be used to increase the number of samples to train and test the models. This can alleviate model overfitting and improve the generalization ability of the trained model. The data augmentation is divided into three different categories: geometric transformations, color space transformations, and pixel point operations.

**Figure 2:**
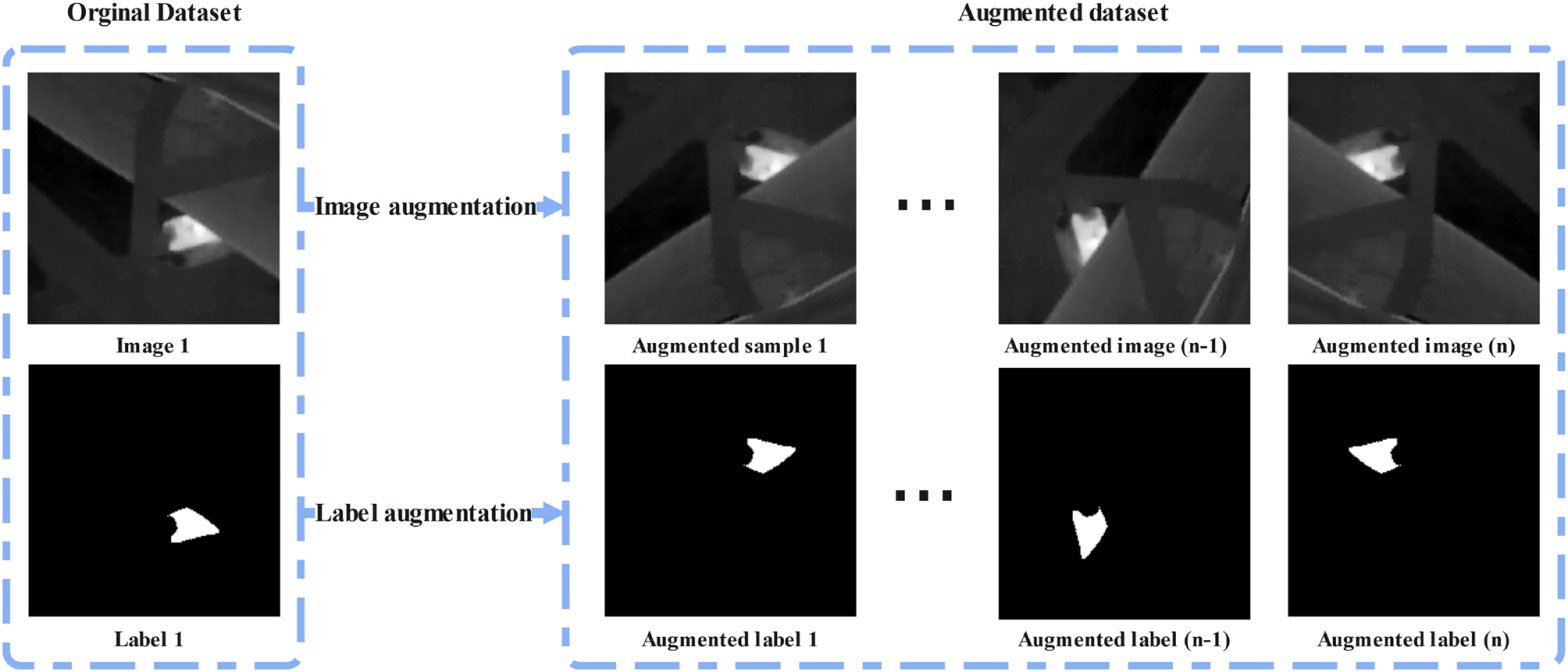
Data augmentation on a thermal image sample and corresponded ground truth label.

In this paper, image augmentation was implemented using the open-source Albumentations package^33,68^. The combination of different data augmentation techniques, namely: vertical flip, random rotation in 90-degree, horizontal flip, and transposes, have been applied to thermal image data sets Fig. 2.

### U-Net architectures for thermal defects detection

The base U-Net architecture was first introduced by Ronneberger et al.^69^ for the automatic segmentation of biomedical images. The U-shaped CNN architecture consists of an encoder–decoder scheme where the encoder is responsible for reducing the spatial dimensions in each layer while increasing the channels. Each encoder block consisted of 3 $\times$ 3 convolutions, where each convolution was followed by a rectified linear unit (ReLU) activation function. In U-Net-based networks, the ReLU is responsible for introducing non-linearity that could increase the generalization of the training data. The output of each ReLU is used as a skip connection to the corresponding decoder block. The decoder is responsible for doubling the spatial dimensions while halving the number of feature channels.

The CNN-based architectures are mostly designed to classify the whole image into a pre-defined category. However, U-Net architectures can provide pixel-level information that enables researchers to analyze target regions with more accuracy. The U-Net architectures have been proven to be a practical tool for semantic segmentation of different images, as they can produce highly accurate segmentation maps using very limited training samples. The limited access to the number of available samples in different fields of study can be crucial, as properly labeled images might not be easily accessible due to the nature of the research.

#### Base U-Net

**Figure 3:**
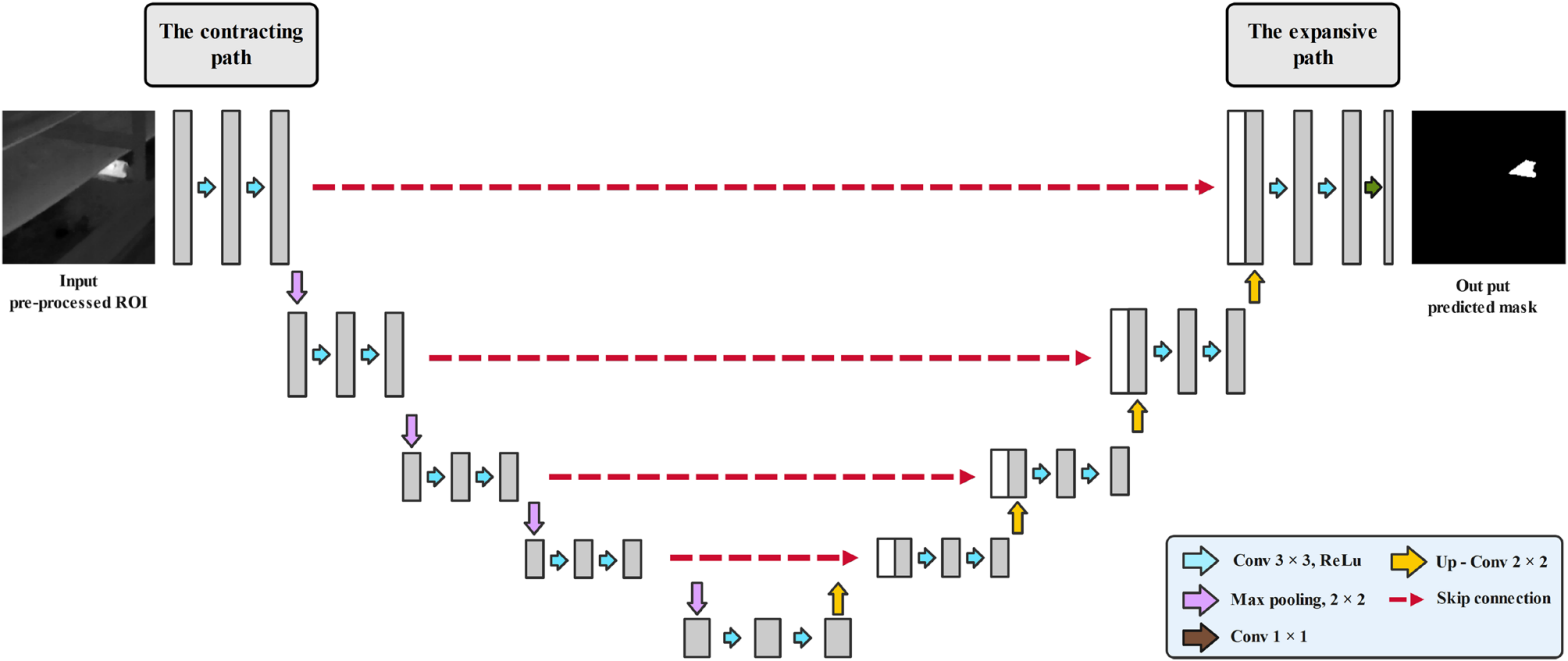
Base U-Net architecture.

The base U-Net network architecture is defined in two parts, where, in the initial phase, a typical CNN architecture is used as a contracting path. In this part, each block is included by two successive $3 \times 3$ convolutions, which are completed by an ReLU activation unit and a max-pooling layer. The expansive path, or second part of the network, is considered the novel idea that was proposed through the base U-Net by Ronneberger et al.^69^. Through this part, the feature map is up-sampled by the $2 \times 2$ up-convolution within each stage. Moreover, the feature map in each corresponding layer in the contracting path is cropped and concatenated into the up-sampled feature map, followed by two successive $3 \times 3$ convolutions and ReLU activation. At the final stage, the feature maps are reduced into the required number of channels, and the output is produced as the desired segmented image. Figure 3 illustrates the network architecture of the base U-Net.

#### Attention U-Net

**Figure 4:**
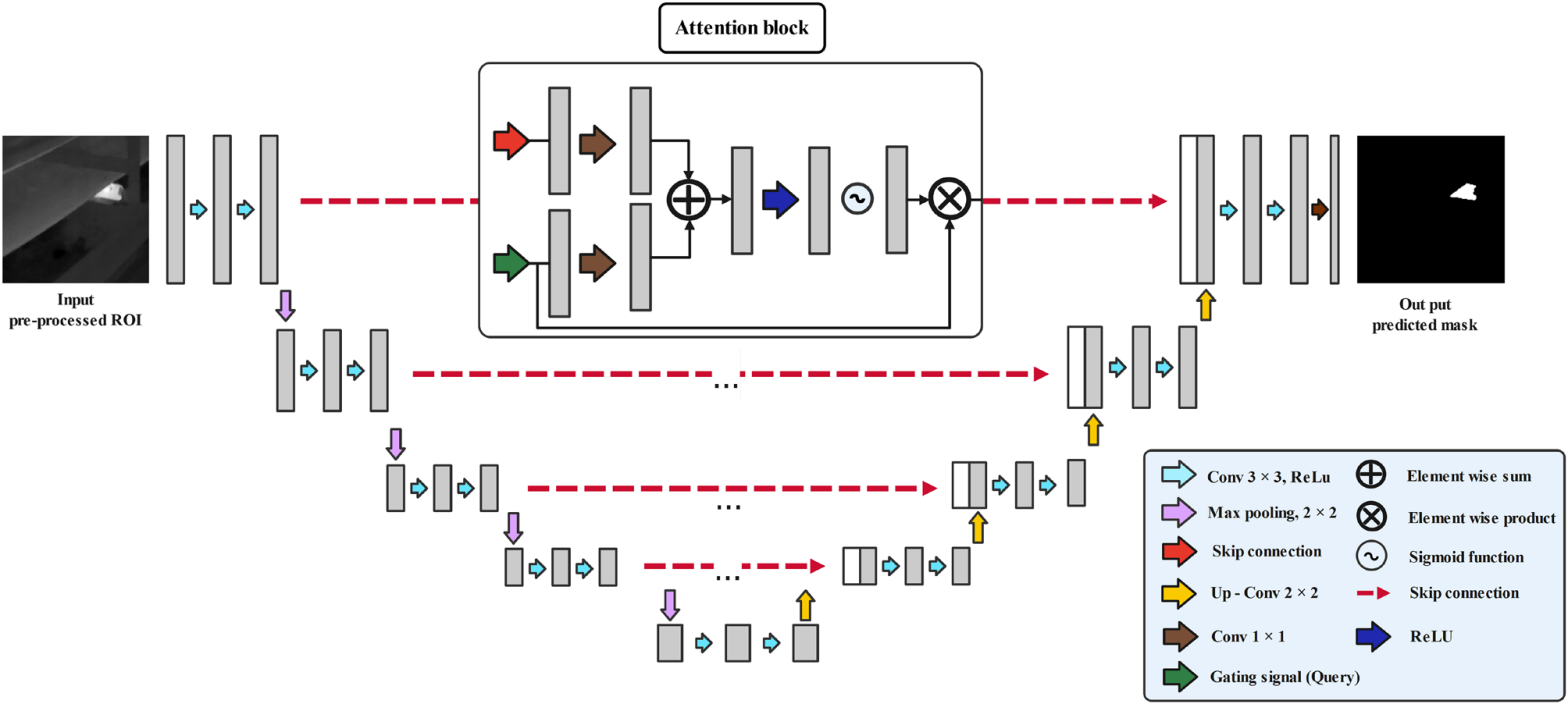
Attention U-Net architecture.

The improved version of the base U-Net architecture was first introduced by Oktay et al. as attention U-Net in 2018^70^. The attention U-Net architecture, Fig. 4, improves the network’s performance in the segmentation of target objects by focusing the network’s attention on specific objects that are desirable to be segmented while ignoring unnecessary areas in input images by making use of the attention gate. The attention gates are in charge of trimming features that aren’t necessary for performing the segmentation task. It is proven that repeated uses of the attention gate after each layer can significantly improve network performance.

Throughout the attention gate, the input signals *g* and $x_{l}$ are passing through a $1\times 1\times 1$ convolution layers. Afterward, the signals are added and undergo a series of linear transformations, including ReLU activation, $1\times 1\times 1$ convolution, sigmoid activation, and an optional grid resampler. At the final level, the original input is concatenated with the output from the sigmoid unit or the resampler.

#### ARes U-Net

**Figure 5:**
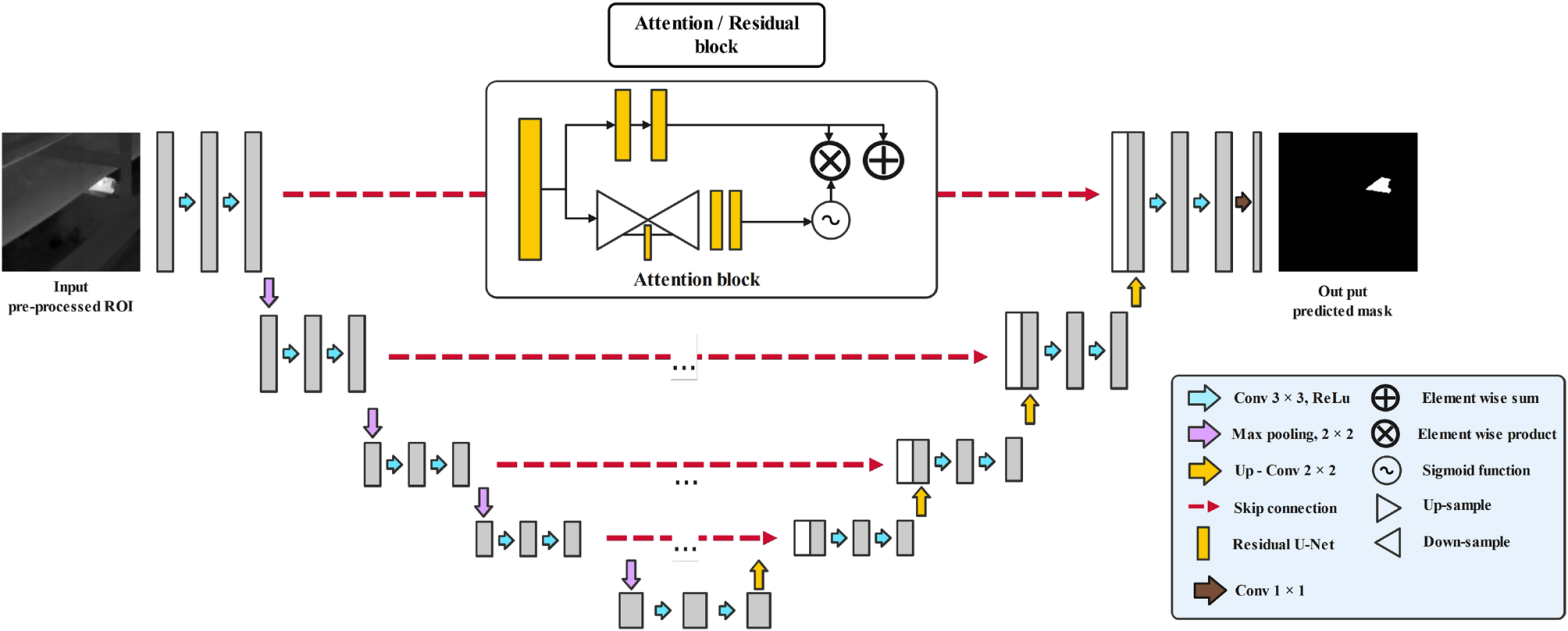
ARes U-Net architecture.

In ARes U-Net^71^, the attention mechanism and residual blocks are embedded into the base U-Net architecture Fig. 5. Therefore, the attention blocks are employed as skip connections to enable the network to focus on desirable regions in the coarse features from the encoder side. Moreover, residual blocks are used to replace the initial convolutional layers to improve the depth of the U-Net network and reduce the chance of gradient vanishing. An exemplary residual block can be defined as follows: *x* and *y* are input and output, respectively, and *F* is an arbitrary learnable function^72^.

3 $$\begin{aligned} y=F(x)+x \end{aligned}$$

The residual can improve the U-Net in semantic segmentation tasks in several different ways. First, to calculate the output parameter *y*, the residual block only needs to learn the residual information, while in the traditional convolutional network, a full mapping between inputs and outputs needs to be calculated, which is a more difficult process. Moreover, the U-Net model can learn to zero out the residuals for producing an identity mapping since the network can decide to select only a subset of the layers when it needs to set some of the layers to be identity mappings.

**Figure 6:**
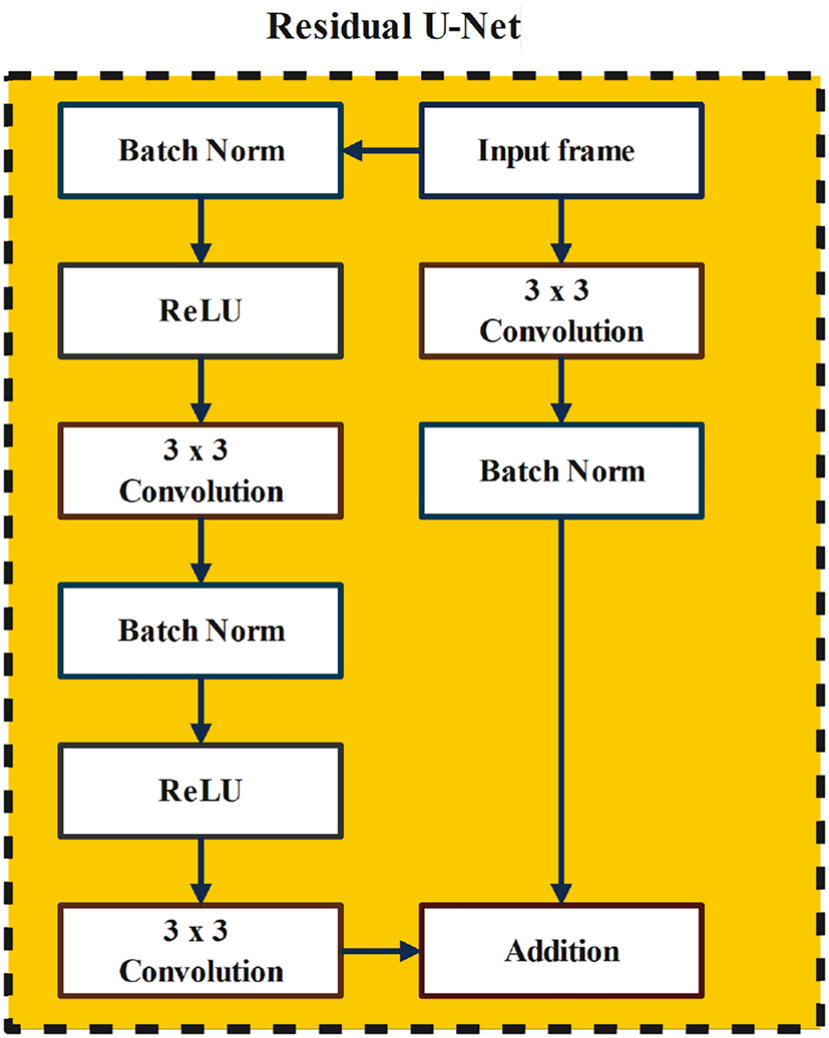
The simplified residual block diagram.

The Resblocks architecture is described in Fig. 6. Each resblock in the studied U-Net model consists of a batch normalization, a rectified linear unit (ReLU), and a convolution. The batch normalization is responsible for simplifying the training process by down-scaling the size of the input. Furthermore, the left side of the network transforms the input image through a series of convolutions and nonlinear activations. Afterward, different paths are added together to let the base U-Net model learn subtle transformations without having to remember the entire image^73–76^.

## Data acquisition

**Figure 7:**
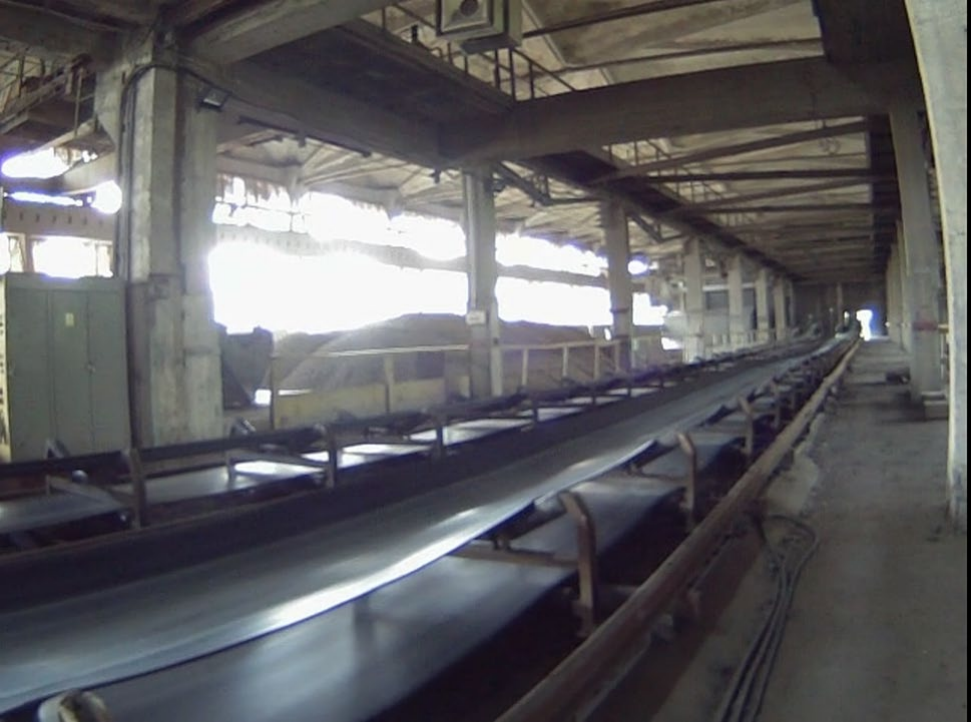
A general picture of the raw materials storage with BC to transport raw materials.

In this paper, we used data sets of thermal videos that were captured from two BC systems Fig. 7. The thermal image datasets numbers one and two were captured from BC system number one. Through the first two experiments, the inspection mobile robot moved forward (FW) and backward (BW) and captured thermal videos from the studied BC system. Through the third experiment, the inspection mobile robot moved forward and captured thermal videos from BC system number two. It is worth mentioning that all videos were captured from the left side of the studied BC systems. Furthermore, all the videos were captured using a FLIR T640 camera with a 45-degree field of view. The format of the captured videos was $768 \times 584$ pixels, 16-bit-colored videos.

**Figure 8:**
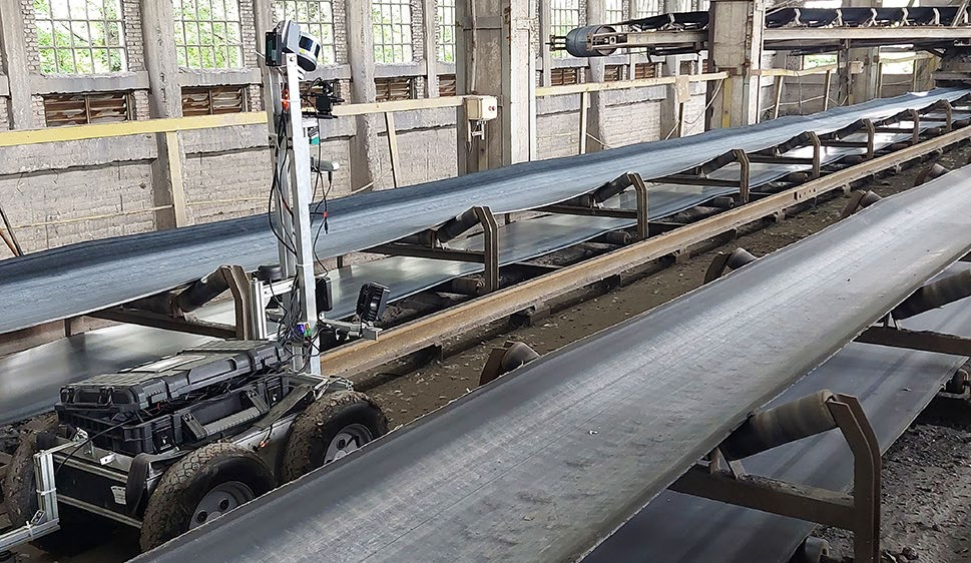
View of the robot during inspection.

The mobile inspection robot in this study is specially designed for the Wrocław University of Science and Technology as a platform that can conduct inspection missions in harsh environmental conditions with a maximum payload capacity of 75 kg. The mobile robot has been manually controlled by a human operator through the experiments Fig. 8.

There are two main approaches for analyzing the thermal condition of objects using thermal cameras. The first can be considered a quantitative analysis, where the exact temperature value of the studied objects should be measured. This measurement can be considered relatively difficult as determining the real and accurate temperature value of the studied object is related to the true emissivity, which should be defined before performing thermal imaging. The second approach is qualitative measurement. In this approach, the relative temperature value of a particular hotspot with respect to another object in a similar environment is measured. In this study, we have chosen qualitative measurement for identification of the thermal defects^77–80^.

Different parameters, such as environmental conditions and the emissivity values of the studied objects, should be considered for precise thermal imaging. In opencast mining sites, solar radiation can be considered a factor that might warm up the BC system components. We conducted our experiments on a sunny day. However, in our case, the solar radiation was mostly blocked by ceiling structures. As long as the idlers needed to be regularly inspected, we tried to provide a solution that could be applicable in different weather and environmental conditions^11,36^.

**Figure 9:**
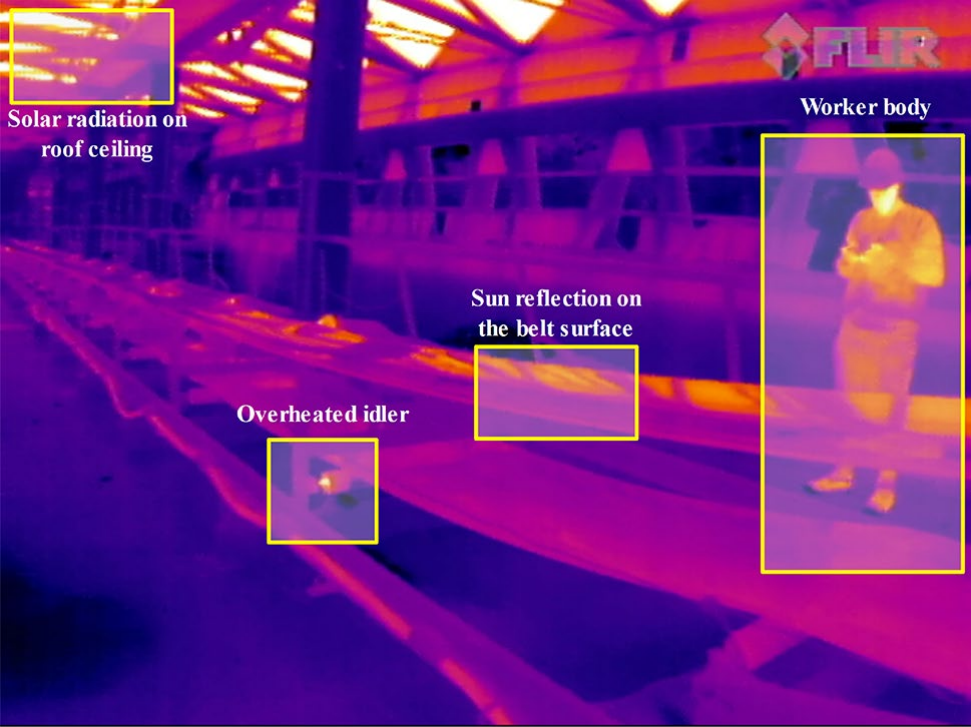
View of the inspection BC system while different thermal sources were presented in captured scene.

**Figure 10:**
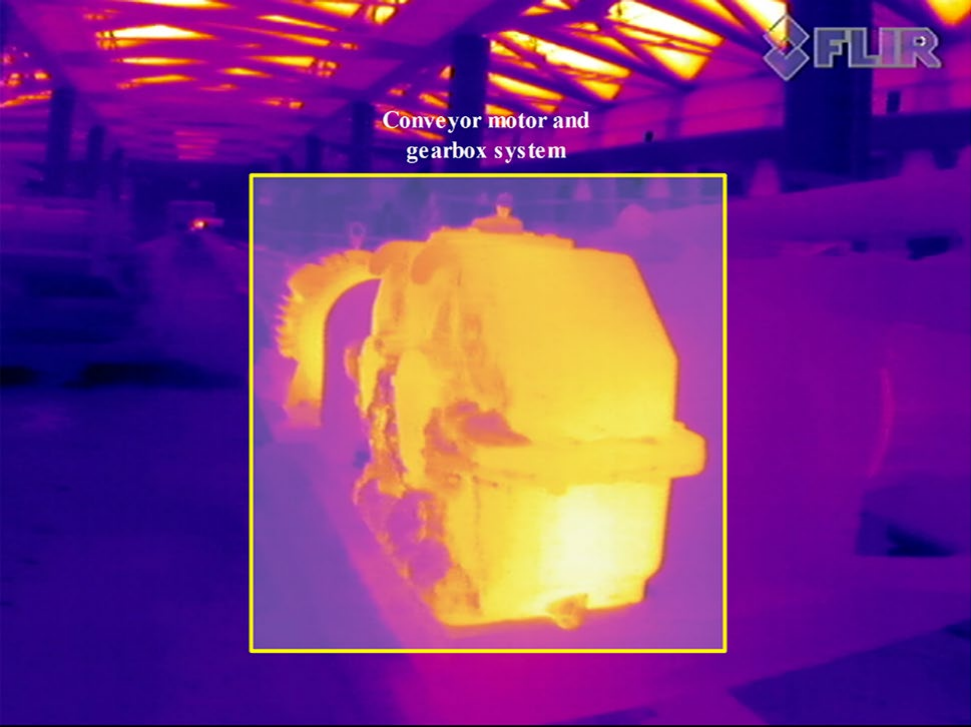
View of the inspected BC system motor and gearbox system that were source of radiation.

Below, we presented different examples of other thermal sources in the studied open-cast mining site that have been captured by a thermal camera and might be wrongly identified as true positive cases (overheated idlers) in Figs. 9 and 10 (FLIR thermal studio suite was used to view and post-process the thermographic results (https://www.flir.eu/products/flir-thermal-studio-suite/)). As long as most of the classical segmentation techniques cannot distinguish between false positives (other thermal sources) and true positives, we proved that the application of deep learning-based segmentation methods can address this issue.

## Training process

Table 1 shows the number of frames extracted from captured thermal videos by the inspection mobile robot. One can notice that the percentage of positive samples in three different experiments was below 7% while the percentage of negative samples was above 93%. The significant difference between the percentage of positive and negative samples can add bias to the training model by skewing the data sets. The imbalanced data set can reduce the performance of the U-Net models in the correct segmentation of overheated idlers.

**Table 1:** Comparison of positive and negative samples extracted from captured thermal videos.

	BC 1 (FW)	BC 1 (BW)	BC 2 (FW)
Total number of extracted frames	6135	6275	10897
Percentage of positive cases	$5.21\%$	$6.67\%$	$5.74\%$
Percentage of negative cases	$94.78\%$	$93.32\%$	$94.26\%$

To ensure that the model has access to a balanced data set, we used data augmentation to increase the number of positive cases. Initially, 10 faulty idlers have been identified and selected from the analyzed data sets. Moreover, we selected 40 frames that contain the faulty samples. After labeling the pixels associated with overheated idlers in each sample, we made a single data set consisting of 400 faulty frames. To increase the number of positive samples and address the class imbalances, each sample undergoes the proposed augmentation and is 10 times oversampled. Therefore, after augmentation process, a unified data set consisting of 8000 samples with equal number of positive and negative samples has been used to train the models.

80% of the data is selected for training and the remaining 20% is used for testing. For training and testing of the studied U-Net architectures, the experiments have been done on the Google Cloud Platform using the NVIDIA A100 with 40 Gb of RAM as the GPU and a 3.8 GHz Intel Xeon CPU with an 8-core on Linux system that has access to 85 Gb of RAM. In this study, the OpenCV library has been used for performing image processing tasks, with Python 3.9 as the programming language and Tensorflow and Keras as the deep learning frameworks.

The studied U-Net models have been trained on the selected training and test sets for 40 epochs. The size of the input images to models was set to $256 \times 256$ pixels, while the batch size was set to 8.

The performance of different U-Net models with the Adam optimizer and BCE as a loss function is measured. BCE loss function is usually employed to train U-Net models for binary classification tasks. In BCE, the loss, or, in other words, error, is a number between 0 and 1, where 0 indicates a perfect model. The BCE loss function equation can be seen as follows:

4 $$\begin{aligned} BCE=-\frac{1}{N} \sum _{i=1}^N y_i \cdot \log \left( p\left( y_i\right) \right) +\left( 1-y_i\right) \cdot \log \left( 1-p\left( y_i\right) \right) \end{aligned}$$

where in Eq. (4), *y* is a label and *p*(*y*) is the predicted probability of a label for all pixel *N*^81^.

## Performance metrics

Different criteria have been proposed to assess the accuracy of different semantic segmentation techniques. Pixel accuracy and Intersection over Union (IoU), also known as Jaccard Index, are the most popular metrics that are currently used by researchers to measure how well models can perform the per-pixel labeling tasks. To explain the metrics, first we assume a total of $k+1$ classes, including a void class or background from $L_{0}$ to $L_{K}$. Moreover, we defined $p_{ij}$ as the number of pixels in class *i* inferred to belong to class *j*. In this direction, we can define $p_{ii}$ as the number of true positives, while $p_{ij}$ and $p_{ji}$ interpreted as false positives and false negatives, respectively^82,83^.

The pixel accuracy metric is simply defined by calculating the ratio between the properly classified pixels and the total number of pixels, as defined below:

5 $$\begin{aligned} \text {Pixel accuracy}=\frac{\sum _{i=0}^k p_{i i}}{\sum _{i=0}^k \sum _{j=0}^k p_{i j}} \end{aligned}$$

The mean pixel accuracy can be defined to calculate correctly classified pixels on a per-class basis and then average over the total number of classes as below:

6 $$\begin{aligned} \text {Mean pixel accuracy}=\frac{1}{k+1} \sum _{i=0}^k \frac{p_{i i}}{\sum _{j=0}^k p_{i j}} \end{aligned}$$

The Jaccard index is a standard metric to measure the performance of the models for performing segmentation tasks. It is working based on calculating the intersection and union of two sets, in our case, the ground truth and our predicted label. The ration can be defined as the number of true positives (TP), or, in other words, the intersection over the sum of TP, false positives (FP), and false negatives (FN) as follows:

7 $$\begin{aligned} \text {Jaccard index} =\frac{T P}{T P+F P+F N} \end{aligned}$$

The mean Jaccard index can be defined as the per-class basis and then averaged as defined below:

8 $$\begin{aligned} \text {Mean Jaccard index}=\frac{1}{k+1} \sum _{i=0}^k \frac{p_{i i}}{\sum _{j=0}^k p_{i j}+\sum _{j=0}^k p_{j i}-p_{i i}} \end{aligned}$$

## Results and discussion

The main reason to define the validation data set is to optimize the hyperparameter values. Ideally, to analyze different models and find the effectiveness of each, they need to be studied regardless of the particular data sets used for validation^84^. As we did not intend to optimize the selected models in this work, important hyperparameters that define the model architectures were adopted from the reference studies, so we only used the train and test data sets to compare the model performances. Moreover, regarding the parameters that define the training process, including the number of training iterations and batch size, we selected the same values for all the studied U-Net models.

**Figure 11:**
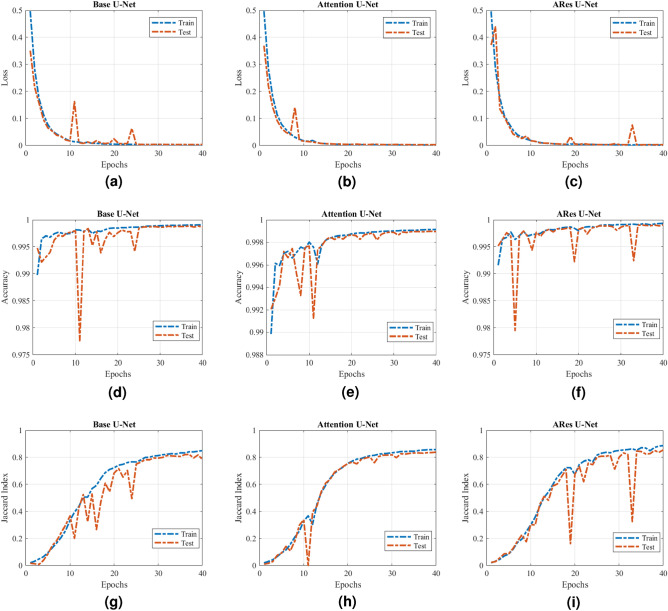
Comparison of performance of studied U-Net models in each training step. (**a**) Loss value (Base U-Net). (**b**) Loss value (Attention U-Net). (**c**) Loss value (ARes U-Net). (**d**) Pixel accuracy (Base U-Net). (**e**) Pixel accuracy (Attention U-Net). (**f**) Pixel accuracy (ARes U-Net). (**g**) Jaccard index (Base U-Net). (**h**) Jaccard index (Attention U-Net). (**i**) Jaccard index (ARes U-Net)

The performance of the studied U-Net architectures with BCE as the loss function over training and test data sets is demonstrated in Fig. 11. The training samples were used to create models that ultimately can produce an accurate result when exposed to new thermal image samples that initially were not used through the training process. On the other hand, the test samples were used to assure the model’s functionality with unseen inputs to simulate real-world scenarios.

Testing and training results from Fig. 11 show that the results of ARes U-Nets have better segmentation performance than other studied models. Moreover, we can see that both base and ARes U-Nets displayed major fluctuations during the test over the test data set, while the Attention U-Net experienced lesser fluctuations, which indicates stability throughout the training process. The relatively stable value of the loss function after 30 epochs of training indicates that any considerable improvement cannot be achieved by extending the training process in the studied models.

The mean values of the Jaccard index and pixel accuracy measures were computed to understand the performance of the studied models. The detailed performance of three different U-Net models over our previous work is summarized in Table 2. The high performance of the different U-Net architectures in performing semantic segmentation tasks has been discussed in different research studies previously. The novel encoder and decoder pathways in U-Net-based CNN reduce computational costs through the training phase. The base U-Net with no modification showed a mean pixel accuracy and a mean Jaccard index of 0.9986 and 0.9080, respectively.

The proposed data set was used to train the other studied U-Net models with different novelties in comparison to the base U-Net network architecture. The attention U-Net was the first modified model that trained on our dataset. The attention gates in this model could modify feature maps to suppress the features in irrelevant areas, which improved the performance of the model in comparison to base U-Net. The attention U-Net performed the second best out of the three models tested in mean pixel accuracy (0.9989) and mean Jaccard index (0.9317). Moreover, the ARes U-Net was employed to perform the segmentation task on our data set. In ARes U-Net, the computation resources are optimized by employing both attention gates and residual blocks. In our study, the ARes U-Net had the best performance in mean pixel accuracy (0.9990) and mean Jaccard index (0.9386).

The computational costs of each model are shown in Table 2. One can notice that the base U-Net had the lowest number of trainable parameters, 31 million (M) with no attention gates or residual blocks, and required the least number of hardware resources and time (40 min) to train.

**Table 2:** Performance comparison of studied methods in segmentation of overheated idlers in thermal images.

Methods	Epoch	Training time	Trainable parameters	Mean Jaccard index	Mean pixel accuracy
Base U-Net	40	40 Min	31M	0.9080	0.9986
Attention U-Net	40	52 Min	37M	0.9317	0.9989
ARes U-Net	40	63 Min	39M	0.9386	0.9990
Siami et al.^36^ (outlier detection method)	–	–	–	0.5687	0.9925

Moreover, the Attention U-Net uses considerably more computational resources (37 M of trainable parameters) and time (52 min) than the base U-Net due to including the attention gates. The increased computational costs of attention U-Net result in slightly higher performance in mean Jaccard index and pixel accuracy compared to base U-Net results. Finally, ARes U-Net with attention gates and residual blocks uses more trainable parameters (39 M) than both U-Net and attention U-Net. ARes U-Net requires the greatest number of parameters while providing a minor improvement in the studied performance metrics. Based on the model metrics, ARes U-Net was the least efficient in terms of computational power.

**Figure 12:**
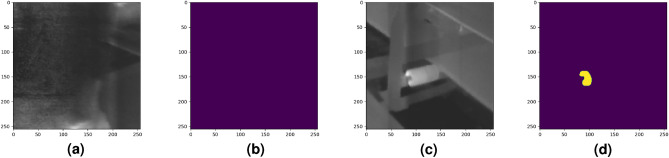
Two selected samples to demonstrate the performance of the studied methods. (**a**) Sample one: Worker body. (**b**) Sample one: Ground truth label. (**c**) Sample two: Overheated idler. (**d**) Sample two: Ground truth label

**Figure 13:**
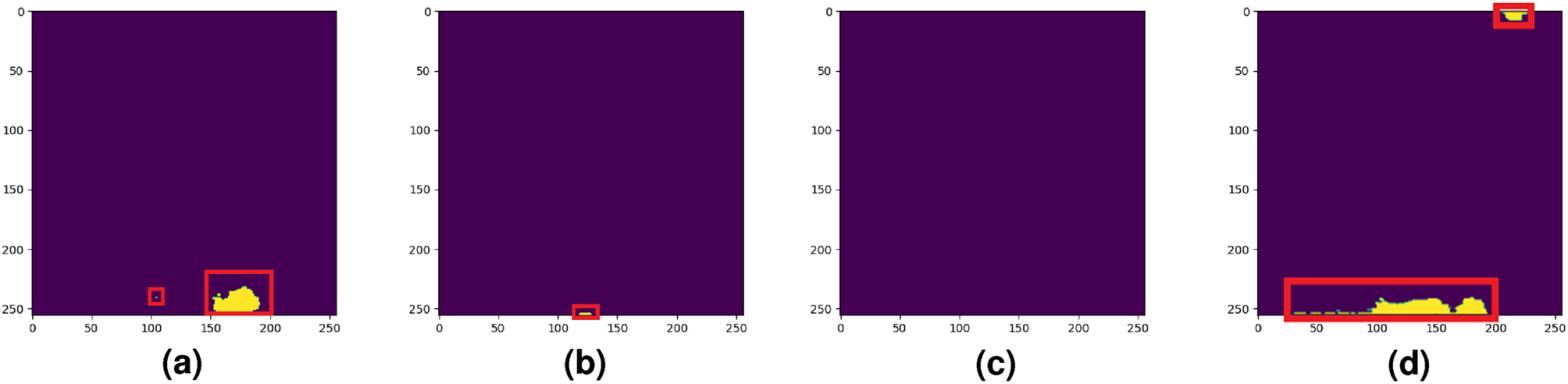
Comparison of overheated idler segmentation results (the red box is the segmentation error)—sample one. (**a**) Base U-Net. (**b**) Attention U-Net. (**c**) ARes U-Net. (**d**) Siami et al.

**Figure 14:**
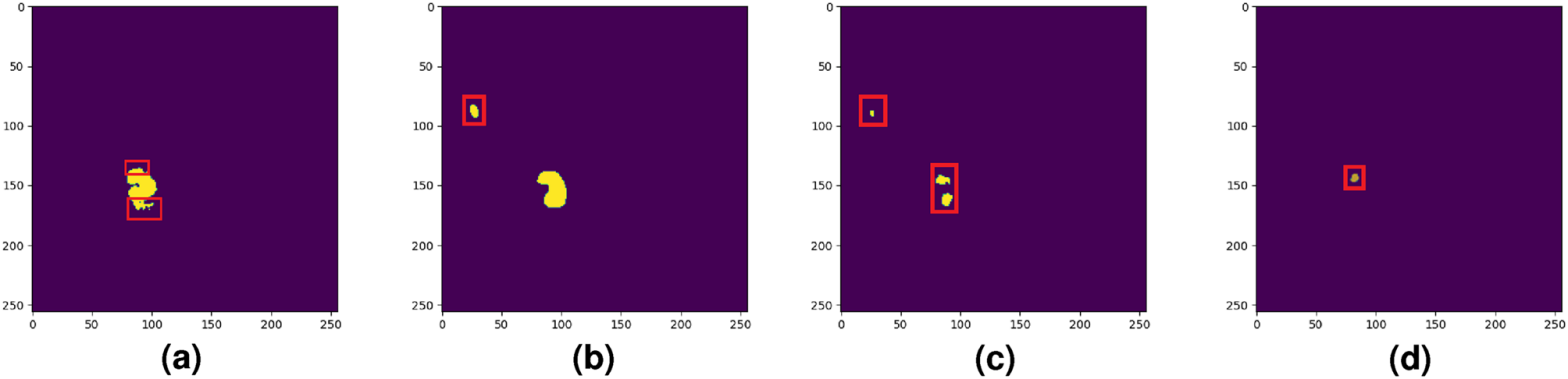
Comparison of overheated idler segmentation results (the red box is the segmentation error)—sample two. (**a**) Base U-Net. (**b**) Attention U-Net. (**c**) ARes U-Net. (**d**) Siami et al.

Furthermore, in Figs. 12, 13 and 14 we compared the performance of the studied deep learning models with our previous study^36^. As we can see from Table 2 and Fig. 13 our previous method caused over-segmentation in thermal images with complex backgrounds, resulting in the mean Jaccard index and mean pixel accuracy of 0.5689 and 0.9925, respectively. It is worth mentioning the high value of mean pixel accuracy does not necessarily indicate the good segmentation performance of our previous study, as it might be highly influenced by imbalanced class data sets^85^.

In Fig. 12, we study the performance of the studied models in two selected samples. Here, we show a special sample where a worker’s body was captured by the thermal camera during the inspection task. This case is a good example to show the studied model behaviors in the segmentation of a sample where the overheated pixels are not related to the studied BC idlers. Moreover, in Fig. 14 we demonstrate a rare case where, despite the performance superiority of ARes U-Net over other studied methods, as mentioned in Table 2 it shows a lower accuracy in distinguishing the overheated idler pixels from the background.

## Conclusion

The proposed deep learning approach can provide automated segmentation of overheated BC idlers in thermal images that are captured by mobile robots. The use of mobile robots enables supervisors to perform regular inspection tasks in hazardous environments like mining sites. Using image augmentation techniques could help us improve the size of the training and test data sets, which would improve the overall performance of the studied U-Net models.

Some limitations should be considered for this study. Firstly, the accuracy of the manually segmented ground truth label can directly influence the overall performance of the studied U-Net models, as the hand labeling of the ground truth might be significantly influenced by human judgments. The small portion of pixels represents the overheated idlers in the captured images. In such cases, the boundaries between the cold background and overheated areas might be unclear due to the low resolution of the extracted region of interest. While it might increase the complexity of the data collection and preparation process, improving the resolution of captured ROIs and increasing the size of hand-labeled ground truth might improve the overall performance of the studied methods.

One complicated factor in this work is the unpredictability of the degree of complexity of the captured thermal images. It can be considered that for the less complicated scenes, the number of required samples for successful training of the studied U-Net architectures may be considerably fewer since each image will contain fewer targets that need to be accurately detected and segmented from each other. Therefore, in such cases, splitting data sets into smaller groups can be an advantage.

Future work will study the tradeoffs between training a single-base U-Net architecture on a large heterogeneous data set in comparison to several smaller homogenous classes. The performance of modern encoder-decoder architectures, such as the different U-Net architectures studied in this study, appears to be robust for image processing tasks. Since the prediction can be performed in milliseconds, it can be considered a proper solution for real-time CM tasks.

## Data Availability

The datasets generated and/or analysed during the current study are not publicly available due to the NDA agreement signed by the authors but are available from the corresponding author on reasonable request.
